# Hemodynamic study of blood flow in the aorta during the interventional robot treatment using fluid–structure interaction

**DOI:** 10.1007/s10237-023-01737-y

**Published:** 2023-06-17

**Authors:** Zongming Zhu, Suqiang Ji, Liang Liang, Hao Wang, Haoyu Xia, Puhua Tang

**Affiliations:** 1 College of Electromechanical Engineering, Changsha University, Changsha, 410022 China; 2 School of Mechanical Engineering and Mechanics, Xiangtan University, Xiangtan, 411105 China; 3 College of Engineering and Design, Hunan Normal University, Changsha, 410081 China; 4 Changsha Institute of Mining Research Co., LTD, Changsha, 410012 China

**Keywords:** Vascular interventional robot, Fluid–structure interaction (FSI), Hemodynamics, Computational fluid dynamics (CFD), Particle image velocimetry (PIV)

## Abstract

An interventional robot is a means for vascular diagnosis and treatment, and it can perform dredging, releasing drug and operating. Normal hemodynamic indicators are a prerequisite for the application of interventional robots. The current hemodynamic research is limited to the absence of interventional devices or interventional devices in fixed positions. Considering the coupling effect of blood, vessels and robots, based on the bi-directional fluid–structure interaction, using the computational fluid dynamics and particle image velocimetry methods, combined with the sliding and moving mesh technologies, we theoretically and experimentally study the hemodynamic indicators such as blood flow lines, blood pressure, equivalent stress, deformation and wall shear stress of blood vessels when the robot precesses, rotates or does not intervene in the pulsating blood flow. The results show that the intervention of the robot increase the blood flow rate, blood pressure, equivalent stress and deformation of the vessels by 76.4%, 55.4%, 76.5%, and 346%, respectively. The operating mode of the robot during low-speed operation has little impact on the hemodynamic indicators. Using the methyl silicone oil as the experimental fluid, the elastic silicone pipe as the experimental pipe, and the intervention robot having a bioplastic outer shell, the velocity of the fluid around the robot is measured on the developed experimental device for fluid flow field in a pulsating flow when the robot runs. The experimental results are similar to the numerical results. Our work provides an important reference for the hemodynamic study and optimization of the mobile interventional devices.

## Introduction

Cardiovascular disease is one of the most serious diseases threatening human beings in the world today. Medication, surgery and interventional therapy are the three main methods to treat the disease. The interventional therapy can achieve clinical effects that drug therapy cannot achieve, and it has the advantages of less pain, less invasion and easy recovery compared to surgical operations, which has become an important method for diagnosing and treating cardiovascular diseases in clinical practice. As one of the devices of the interventional therapy, a vascular interventional robot can enter blood vessels, and move freely in the vessel to perform tasks such as diagnosing blood vessels, unblocking blood vessels and delivering drugs at a fixed point. When a robot intervenes in the blood vessel, the deformation of the blood vessel, the flow characteristics of the blood and the motion of the robot have a coupling relationship. Only by deeply studying the flow characteristics of the blood during the movement of the robot can we evaluate the hemodynamic indicators and safety during the intervention and treatment, and further optimize the structure and operating parameters of the robot within the scope of the safe treatment.

Since the hemodynamic factors are closely related to the effectiveness of the cardiovascular disease treatment, it is necessary to introduce the computational fluid dynamics (CFD) and fluid–structure interaction (FSI) to predict these hemodynamic factors before deciding on the diagnosis and treatment plan. The CFD method is mainly used to solve fluid problems, such as fluid flow resistance, velocity and pressure, and its calculation process only considers the fluid motion. The FSI method considers the solid (structure) deformation while analyzing the fluid motion, and it solves the problem based on the fluid–solid coupling effect. The CFD method was used to study the differences in hemodynamics of carotid arteries undergoing primary closure or stent placement (Jung et al. 2022). Rahma and Abdelhamid (2023) explored the hemodynamic parameters of cerebral aneurysms and their impact on aneurysm rupture, and analyzed the effect of the flow recirculation on the hemodynamic parameters of the convex wall. Mazo et al. (2022) numerically simulated the oscillatory flow of a viscous incompressible fluid in a rigid round pipe with local constrictions. The above hemodynamic studies were conducted using the CFD method. It is assumed that the vessel walls are rigid walls in the numerical simulation and the blood vessels as elastic materials are not considered. In order to make the numerical results more consistent with the blood flow in real blood vessels, the elasticity of the blood vessels should be considered, and the interaction between blood vessels and blood flow should be taken into account, namely, the fluid–structure interaction exists between the blood flow and blood vessel deformation. Therefore, the numerical calculation using the fluid–structure interaction method can better reflect the changes in the hemodynamic indicators within blood vessels.

Currently, the research on hemodynamics mainly focuses on whether there is an interventional device or whether the interventional device is in a fixed position. For these situations, researchers have conducted a large number of numerical calculations and experimental studies. The effect of geometric parameters of coronary arteries on hemodynamic indicators such as low wall shear stress (WSS) and local disturbance flow were numerically investigated, and it was found that the blood flow in the right ventricular branch of the lower curvature, smaller cross sectional area and higher angles is conducive to the formation of atherosclerosis (Pinho et al. 2019). Kumar et al. (2022) studied normal and specific stenotic carotid artery models with the fluid–structure interaction, and found that the hypertension increases the vascular peripheral resistance, which in turn decreases WSS. Ma et al. (2022) numerically analyzed the effect of stenosis rate and location on plaque development in vertebral arteries. The results showed that the flow velocity and WSS at the stenosis are increased with the degree of stenosis, and the plaque shedding is also increased. The blood flow characteristics of the aorta with aortic dissection was evaluated by using the finite element analysis and fluid–structure interaction, taking into account the deformation of aortic walls (Keramati et al. 2020). The effect of the aneurysm geometric features on the hemodynamic characteristics within a left coronary artery was analyzed by the numerical method (Rafiei and Saidi 2022). The fluid–structure interaction method was used to analyze different models of intracranial vessels, and three models of normal arteries, atherosclerotic arteries and aneurysmal arteries are established (Rostam-Alilou et al. 2022). The results indicate that the occlusion of the arterial cross section plays a decisive role in altering the hemodynamic behavior of the arteries. The adverse changes in blood flow velocity and pressure throughout the entire vessel increase the risk of arterial endothelial tissue remodeling and aneurysm formation. Blood itself is an elastic non-Newtonian fluid. In order to simplify the calculation, blood properties are often idealized and studied as incompressible Newtonian fluid. Mendieta et al. (2020) compared the wall shear stress and blood pressure gradient under the Newtonian model of blood and four non-Newtonian models (Carreau, Cross, Quemada, and Power-law) by the numerical analysis. The results showed that the assumption of the Newtonian model is reasonable when studying the overall flow pattern or time-averaged wall shear stress of blood. In addition, the blood flow in the human arteries is pulsatile in nature. The effects of pulsatile and uniform inlet flows on the hemodynamics of the abdominal aorta bifurcation were compared by the numerical simulation (Soares et al. 2021). Differences in the magnitude of blood velocities can also cause the changes in hemodynamic indicators. Wu et al. (2022) studied the wall deformation, pressure and shear stress characteristics of human arm arteries under the boundary conditions for different inlet velocities. The above researches were conducted without interventional devices, and the quantitative analysis of hemodynamic indicators was conducted by the numerical simulation. The blood flow characteristics and the relationship between blood flow and vessel wall deformation were studied, which reflects the health status inside blood vessels.

With the continuous progress of medical technology, the interventional therapy has been widely used in clinical applications. At present, the interventional therapy has become the main treatment method for many diseases, especially in fields such as cardiovascular diseases, tumors, and so on. When an interventional device is implanted into a blood vessel, the internal structure of the blood vessel is changed, and blood flow is also changed accordingly. Scholars have studied the hemodynamics of blood flow inside blood vessels when there are interventional devices. The hemodynamic parameters of overlapping bare-metal stents intervention for treating aortic aneurysms were studied (Zhang et al. 2014). They found that the stent intervention induces a low-speed flow field near the aneurysm wall. The overlapping stents intervention will cause a significant decrease in the wall shear stress and a slight reduction in the pressure acting on the aneurysm wall, and the distribution of the pressure becomes more uniform. Dai et al. (2020) explored the effect of multiple overlapping uncoated stents (MOUS) on the hemodynamics of the aortic dissection. The results showed that the use of MOUS with low porosity slows down the blood flow within the false lumen and reduces the wall shear stress. Polanczyk et al. (2021) presented a non-invasive method of mass flow rate/velocity and wall stress analysis in type B aortic dissection by using the computer tomography angiography (CTA) and computational structural analysis. Liu et al. (2022b) combined the finite element method and CFD method to investigate the hemodynamic mechanism during the plug-in coil embolization in the treatment of venous diverticulum. A non-Newtonian suspended particle model was proposed to simulate the real blood flow, and the local hemodynamic characteristics of the artery containing the stent were further investigated (Wang et al. 2021). The results showed that there is a flow stagnation zone when the stent strut protrudes into the stent, and the stent implantation causes the uneven pressure gradient distribution. The bi-directional fluid–structure interaction method was used to study the changes in the vessel wall deformation and wall shear stress when the vascular robot was in a fixed position, as well as the effect of the elastic wall on the robot propulsive performance (Jiang et al. 2014). The above hemodynamic studies assume that the interventional device is in a fixed position, and the devices include stents, catheters, guide wires, coils, interventional robots, etc. Robot intervention is currently a highly promising method of vascular therapy, but there is currently no report on the hemodynamic research considering the fluid–structure interaction during the movement of vascular robots.

In order to verify the correctness and feasibility of the numerical calculations, some researchers also performed experimental studies on the intravascular hemodynamics. Li et al. (2023) established a real blood vessel model based on CTA, and investigated the effects of the iliac vein structures with different stenosis rates, taper angles, and left branch inclination angles on the time-averaged wall shear stress, oscillatory shear index and relative resident time by means of the theoretical studies and in vitro experiments. It was found that the iliac vein stenosis significantly increases the wall shear stress of blood vessels in the stenosis and intersection area. As the taper is increased, the risk of the thrombus formation in the low wall shear stress zone is increased. A smaller inclination angle will exacerbate the effects of stenotic vessels on blood flow characteristics and vessel walls. Using swept-source optical coherence tomography angiography (SS-OCTA), the relationship between the retinal microvessels and cerebral hemodynamics in patients was experimentally investigated with internal carotid artery (ICA) stenosis (Liu et al. 2022a). The results indicated that the changes in shallow retinal blood flow perfusion in patients with unilateral moderate or severe ICA stenosis are related to the changes in cerebral hemodynamics. A new ex-vivo system called Human-Cardiovascular-System-Phantom (HCSP) was proposed to simulate pulsatile hemodynamic in the abdominal aortic aneurysm (AAA) before and after stent–graft placement (Polanczyk et al. 2018). The particle image velocimetry (PIV) was used to measure the transient flow patterns in simplified saccular intracranial aneurysm models and the influence of the aspect ratio on the evolution of flow patterns and hemodynamics in intracranial aneurysms was revealed (Shen et al. 2022). Yeh et al. (2019) performed a simulation analysis of the hemodynamics of the bileaflet mechanical heart valve and valve mechanics, and used the particle image velocimetry (PIV) technology to conduct an experiment on an in vitro bench model by a ViVitro pulse replicator. The PIV technology is a popular method in the hydrodynamic measurement, which depends on the tracer particles in the flow field to measure the transient velocity of the fluid. At present, the experimental measurement of hemodynamics mainly uses the medical imaging equipment and PIV technology, and the simulated pulsatile flow is mainly generated by peristaltic pumps or other self-made devices. The numerical simulation and experimental measurement of hemodynamics complement and confirm each other, which is of great significance.

Currently, the numerical simulation and experimental study on hemodynamics mainly focus on the situation where there is no interventional device or the interventional device is fixed in the blood vessel. The process of the interventional device entering the blood vessel or its movement in the blood vessel has been not considered. There is a lack of comprehensive analysis of the coupling relationship among the blood flow, vessel wall deformation and moving interventional devices. Based on the bi-directional fluid–structure interaction between blood and blood vessels, we conduct the numerical simulation and experimental research on the fluid flow characteristics when an interventional robot is precessing in a pipe filled with pulsatile fluid. This paper lays a foundation for the safe intervention and diagnosis of vascular robots.

## Structure and drive principle of a vascular interventional robot

**Fig. 1:**
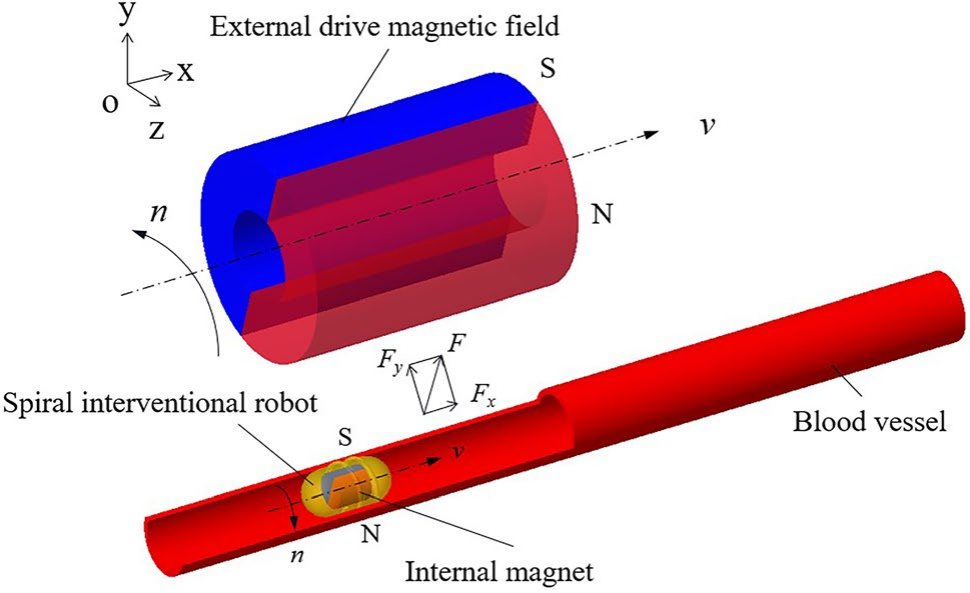
Schematic diagram of the structure and drive principle of a vascular interventional robot

In Fig. 1, the external drive magnetic field is based on a permanent magnet with a hollow circular ring shape and radial magnetization. The vascular interventional robot has a spiral rib on the outer surface and a solid cylindrical magnet with radial magnetization inside, which attracts the external permanent magnet with opposite poles. By controlling the translational and rotational motion (*i.e*., precession) of the external permanent magnet, the spiral robot is driven to make the same precessing motion, thus playing a role in unblocking blood vessels and administering drugs at designated locations within the blood vessels.

## Numerical calculation model and method

### Dynamic control equations

Assuming that the intravascular fluid is temperature independent and incompressible, there are the continuity equation and the momentum conservation equation of fluid (Zhu et al. 2022):

1 $$ \nabla v = 0 $$

2 $$ \rho_{f} \frac{\partial v}{{\partial t}} + \rho_{f} \left[ {\left( {v - d_{f} } \right) \cdot \nabla v} \right] = - \nabla p + \nabla \tau_{f} + F_{f} $$

According to the Newton's second law, there is:

3 $$ \nabla \sigma_{s} + F_{s} = \rho_{s} d_{s} $$

where *τ*_*f*_ is the viscous stress tensor, $\nabla \tau_{f} = \mu \nabla^{2} v$, *µ* is the fluid viscosity, *v* is the fluid velocity vector, $\nabla v = \frac{{\partial u_{x} }}{\partial x} + \frac{{\partial u_{y} }}{\partial y} + \frac{{\partial u_{z} }}{\partial z}$, *v*_*x*_, *v*_*y*_, *v*_*z*_ are the components of the fluid velocity vector *v* in the *x*, *y*, *z* directions, *ρ*_*f*_ is the fluid density, *F*_*f*_ is the volume force acting on the fluid, *p* is the fluid pressure, *d*_*f*_ is the moving boundary velocity vector, *t* is time, *σ*_*s*_ is the vessel wall stress tensor, *d*_*s*_ is the local acceleration in the vessel wall region, *ρ*_*s*_ is the vessel wall density, and *F*_*s*_ is the vessel wall volume force vector.

Equations (1) and (2) are the dynamic control equations of viscous fluid (blood fluid), which are the mathematical models for the numerical calculation of the fluid flow field, and Eq. (3) is the dynamic control equation for solid (vessel structure).

The fluid–structure interaction follows the basic conservation principle, and the following equations are also satisfied on the fluid–solid coupling surface without considering the effects of temperature and heat:

4 $$ \left\{ {\begin{array}{*{20}l} {\sigma_{f} n_{f} = \sigma_{s} n_{s} } \hfill \\ {u_{f} = u_{s} } \hfill \\ \end{array} } \right. $$

where *σ* is the stress tensor, *n* is the boundary normal vector, *u* is the displacement vector, *f* is the subscript denoting fluid, and *s* is the subscript denoting solid.

### System modeling

The vascular robot system includes robot, blood vessel and blood fluid. The total length of the robot is 18 mm, the two ends are semicircular spheres with a diameter of 10 mm, and the middle section is a smooth cylinder with a length of 8 mm and a diameter of 10 mm. The surface of the middle section has semicircular threads with a cross sectional radius of 0.5 mm, a pitch of 1.5 mm, and a thread length of 6 mm. According to the size range of the actual human thoracic aorta, the inner diameter of the selected vessel is 18 mm, the length is 80 mm, and the wall thickness is 1 mm. The real blood vessel is a multi-layer structure, and the constitutive relationship is nonlinear. In this paper, we simplify the blood vessel to a single-layer structure and it is a linear elastic material. Assuming the density of the vessel material is 1.15 × 10^3^ kg/m^3^, the elastic modulus is 1 × 10^6^ Pa, and the Poisson's ratio is 0.45. With reference to the human blood characteristics, the blood density is assumed as 1.053 × 10^3^ kg/m^3^, and the viscosity is 5 × 10^–3^ Pa s.

**Fig. 2:**
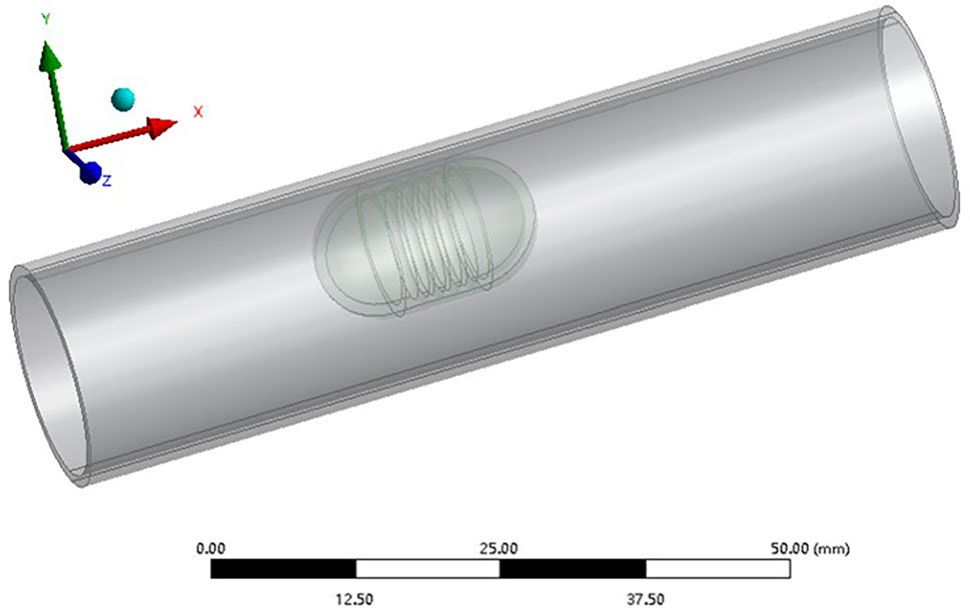
Computational geometry model of the vascular robot system

In order to simulate the motion of the fluid around the robot, a wrapped fluid is designed on the surface of the robot. The shape of the wrapped fluid is similar to the shape of the robot, and the thickness is 0.5 mm. The computational geometric model of the robot system is shown in Fig. 2.

### Grid division

The numerical calculation is carried out using the software Ansys Workbench 2021, and we use four modules: Geometry, Fluid Flow (Fluent), Transient Structural, and System Couple to complete the bi-directional fluid–structure interaction calculation. Due to the use of partition coupling algorithm in the System Couple module, the grids of the solid and fluid need to be independently divided.

**Fig. 3:**
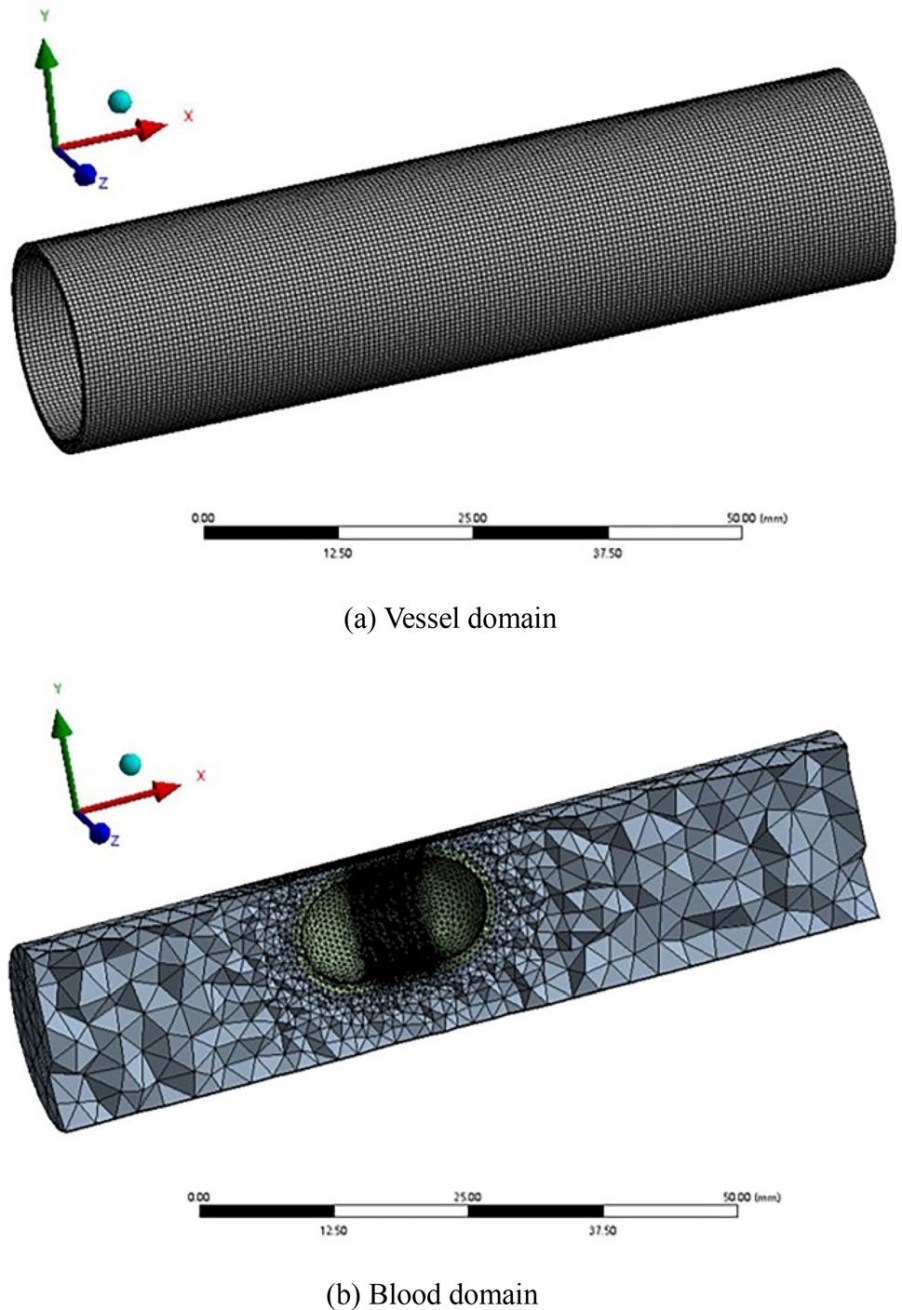
Computational domain grids

The Mesh function in the Model component of the Transient Structural module is used to mesh the blood vessel (solid). The mesh type is Hexahedral, the physical preference is Mechanical, and the solver preference is Mechanical APDL. In Fig. 3a, the grids of the solid domain include 39,680 elements and 219,232 nodes.

The Mesh component in the Fluent module is used to mesh blood (fluid). The method of tetrahedral mesh combined with wedge mesh (boundary layer domain) is mesh irregular fluid zone. The physical preference is CFD, and the solver preference is Fluent. In Fig. 3b, the grids of the fluid domain include 348,224 elements and 102,992 nodes.

### Grid independence analysis

The number of grids has a significant impact on the calculation results. We use the fluid velocity at the vessel outlet during the motion of the robot as a reference indicator for grid independence analysis.

**Fig. 4:**
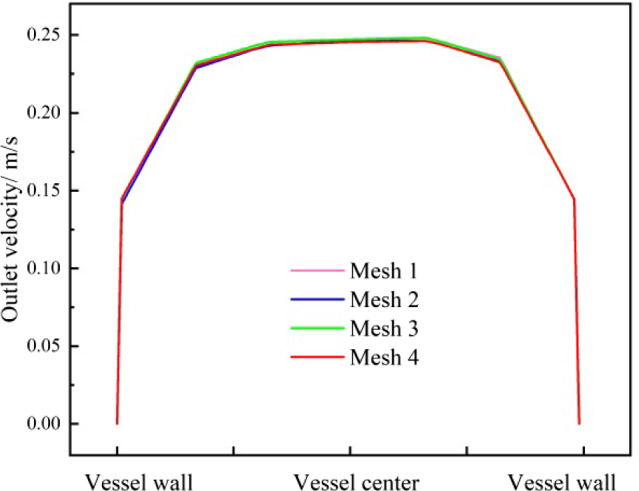
Fluid velocity at the vessel outlet showing mesh independence

According to the above grid division method, the grids are gradually refined and the numerical simulations are performed under the same conditions of other parameters. Four groups of different numbers of grids are used for the numerical simulation in Table 1. The relationship between the number of grids and the fluid velocity at the vessel outlet is obtained as shown in Fig. 4. When the number of grids is increased from 387,907 to 632,711, the change rate of the fluid velocity of the vessel outlet is less than 1%, meeting the requirement of the grid independence. So the number of grids is chosen to be 387,907.

**Table 1:** Details of the mesh independence study

No.	Mesh elements	Mesh nodes	Outlet velocity (m/s)	Difference (%)
1	131,449	248,664	0.16618	–
2	245,007	281,510	0.16340	1.931
3	387,904	322,224	0.16654	1.923
4	632,771	397,130	0.16650	0.026

### Bi-directional fluid–structure interaction calculation method

When a vascular robot moves in a blood vessel, blood pressure will cause the vessel to deform, which in turn affects blood flow. Therefore, a bi-directional fluid–structure interaction method will be needed to solve the problem. The fluid calculation uses the CFD method by the Fluid Flow (Fluent) module, and the solid calculation uses the (computational solid mechanics, CSM) method by the Transient Structural module. The data transmission, coupling conditions, and other settings are then made by the System Couple module.

**Fig. 5:**
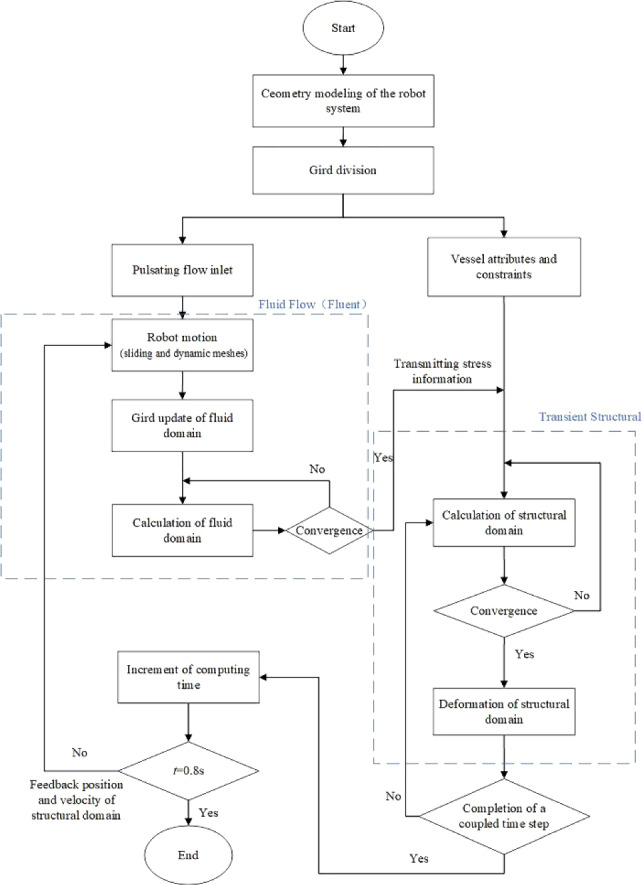
Flowchart of the numerical calculation

The fluid domain (blood) and the structural domain (vessel) are solved in parallel. In the each sub-step, both the fluid domain solution and the structural domain solution need to converge before proceeding to the next step, otherwise they need to be recomputed until the results converge. Firstly, the fluid calculation domain is solved, and then the pressure information is transmitted to the solid domain. After receiving the pressure information, the structural domain generates the displacement, which causes the changes in the structural calculation domain, leading to dynamic mesh reconstruction. Then, it is fed back to the fluid domain, and the time step continues to increase until the calculation cycle is reached, thus ending. The entire calculation process is shown in Fig. 5.

### Pulsatile blood flow inlet and solution parameter settings

**Fig. 6:**
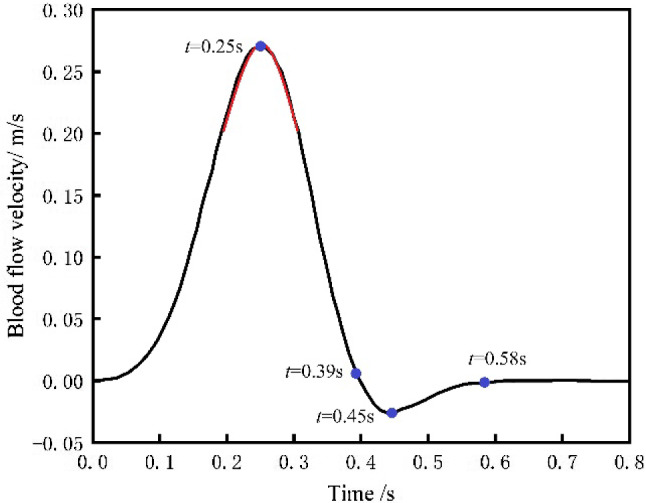
Blood flow velocity at the inlet of the aorta

Due to the regular contraction and relaxation of the atriums and ventricles, the heart achieves the intermittent ejection, resulting in the strong pulsatile characteristics of blood flow in the arteries. The pulsation frequency of the heart is usually 75 beats per minute, or a cycle of 0.8 s. Using the aortic blood flow velocity from the reference (Pedley 1980) (See Fig. 6) as the inlet condition, and the velocity curve is fitted as a function equation of velocity and time (See Eq. 5).

The user defined function (UDF) is used for the inlet condition of pulsatile blood flow. The inlet and outlet faces of the vessel are defined as fixed support conditions with displacement of 0, and the outlet pressure is set to 0. Because of the rotation of the robot, the fluid state in the blood vessel is turbulent, and the standard *k*-*ε* turbulent model is used. The turbulent intensity is set at 5%, and the hydraulic diameter is set at 0.018 m. The fluid flow near the wall is processed by using the standard wall function, and the wall conditions are set to non-slip stationary walls.

5 $$ v_{\text{inlet}} = \left\{ {\begin{array}{*{20}l} { - 0.742t + 16.144t^{2} - 37.289t^{3} } \hfill & {\left( {0 \le t < 0.39} \right)} \hfill \\ {0.183 - 0.477t} \hfill & {\left( {0.39 \le t < 0.45} \right)} \hfill \\ { - 0.109 + 0.188t} \hfill & {\left( {0.45 \le t < 0.58} \right)} \hfill \\ 0 \hfill & {\left( {0.58 \le t \le 0.8} \right)} \hfill \\ \end{array} } \right. $$

According to the actual situation, the translational speed of the robot is set to 4 mm/s in the numerical calculation, the moving direction is opposite to the direction of blood flow, and the rotational speed of the robot is 120 r/min. The standard SIMPLE algorithm is used to solve the coupled equations of fluid pressure and velocity. The differential formats of pressure, momentum, turbulent kinetic energy and dissipation rate are all in the second-order upward format. In order to simulate the motion of the fluid adjacent to the robot, a sliding mesh is used, and the rotational speed of the fluid adjacent to the robot is equal to that of the robot.

The entire numerical calculation is performed in a transient manner. It is assumed that the robot moves in the pipe along the positive direction of the *x*-axis, and its motion law is controlled by the UDF function. The grid update caused by the robot's movement and the fluid–structure interaction of the blood and vessel is needed to be achieved by the dynamic mesh function. During the creation of the dynamic mesh, the interface between blood and vessel is set as the system coupling surface, and the adjacent boundary layer is set to be deformed when the dynamic mesh is reconstructed. In the system coupling module, the blood vessel interface is selected to create the data transfer, and the under-relaxation factor of the force transfer is set to 0.5. The time step in the transient calculation is set to 0.01 s and the end time of the calculation is 0.8 s.

## Numerical calculation results and analysis

Considering the interaction between blood flow and blood vessels, the bi-directional fluid–structure interaction calculation method was used to numerically calculate the hemodynamic parameters in the pulsatile blood flow when the robot is precessing, rotating at a fixed position, or does not exist.

### Blood flow streamlines

**Fig. 7:**
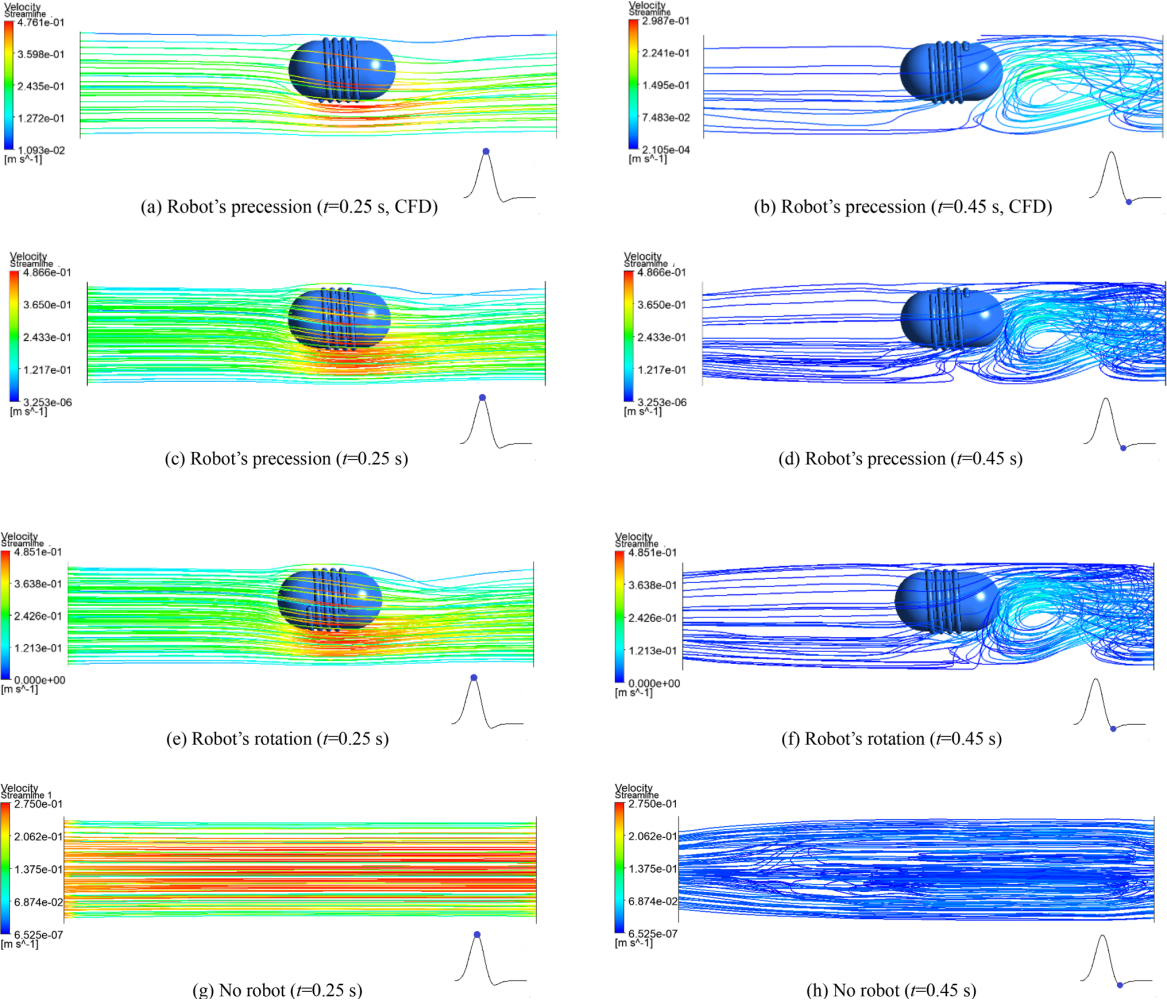
Velocity streamlines of the blood flow under different conditions at 0.25 s and 0.45 s

Figure 7 shows the velocity streamlines of the blood flow at the maximum positive value (*t* = 0.25 s, during systole) and the maximum negative value (*t* = 0.45 s, during diastole) of the blood flow velocities under the three conditions. Blood flows from the left inlet to the right outlet, and the robot moves from right to left. Figure 7a and b show the calculated results using the CFD method (without considering blood vessel deformation), while Fig. 7c–h show the calculated results using the FSI method. The figures show that the blood flow trajectory when using the CFD method is basically the same as that when using the FSI method, but the values are slightly smaller. Considering the real blood vessels are elastic, so the FSI method for the calculation is more reasonable.

At the time of 0.25 s, when the robot is precessing or only rotating, their changing trends of velocities are basically the same, the zones with the fluid maximum velocity are located directly below the robot, and their difference in the maximum velocity values is very small. When there is no interventional robot, the blood flow shows an obvious laminar model, the blood flow streamlines are parallel to each other, and it is close to real blood flow. That is, at different times, there is always the maximum blood flow velocity in the center of the vessel, the blood flow velocity is gradually decreased toward the surrounding vessel walls, and it is decreased to 0 near the wall. The distribution of the blood flow velocity is in a parabola shape at the cross section of the vessel. The intervention of the robot has a significant impact on the blood flow, it increases the blood flow rate by 76.4%, while the impact of the robot operating mode on the blood flow velocity is minimal when the robot is moving at a low speed of 4 mm/s. The intervention of the robot reduces the inner diameter of the blood vessels through which blood flows. When the blood flows below the robot, the fluid zone is compressed, accelerating the flow of blood and forming a high-speed fluid zone, which increases the force of blood on the vessel wall and easily causes damage to vascular endothelial cells. For the entire cardiac cycle, the maximum blood flow velocity is 0.487 m/s, which is within the safe range of the human body and will not cause damage to the blood vessels.

At the time of 0.45 s, the blood flows in the opposite direction and there is a negative velocity value, which is significantly slower than that at the time of 0.25 s. When the blood flows through the robot, it bypasses around the robot and converges at its tail, forming a huge vortex zone behind the robot. The blood flow at this zone changes from laminar to turbulent, and the flow state becomes complex. At the same time, because the flow rate is slow, the blood flow power is insufficient, which may cause pathological changes such as inflammatory reactions and oxidative stress in endothelial cells. However, the robot does not work in a fixed position for a long time, and the negative impact caused by the short-term changes in the blood flow rate during the precession process is minimal, making it is safe and reliable for the diagnosis and treatment process of the interventional robot.

### Blood pressure

**Fig. 8:**
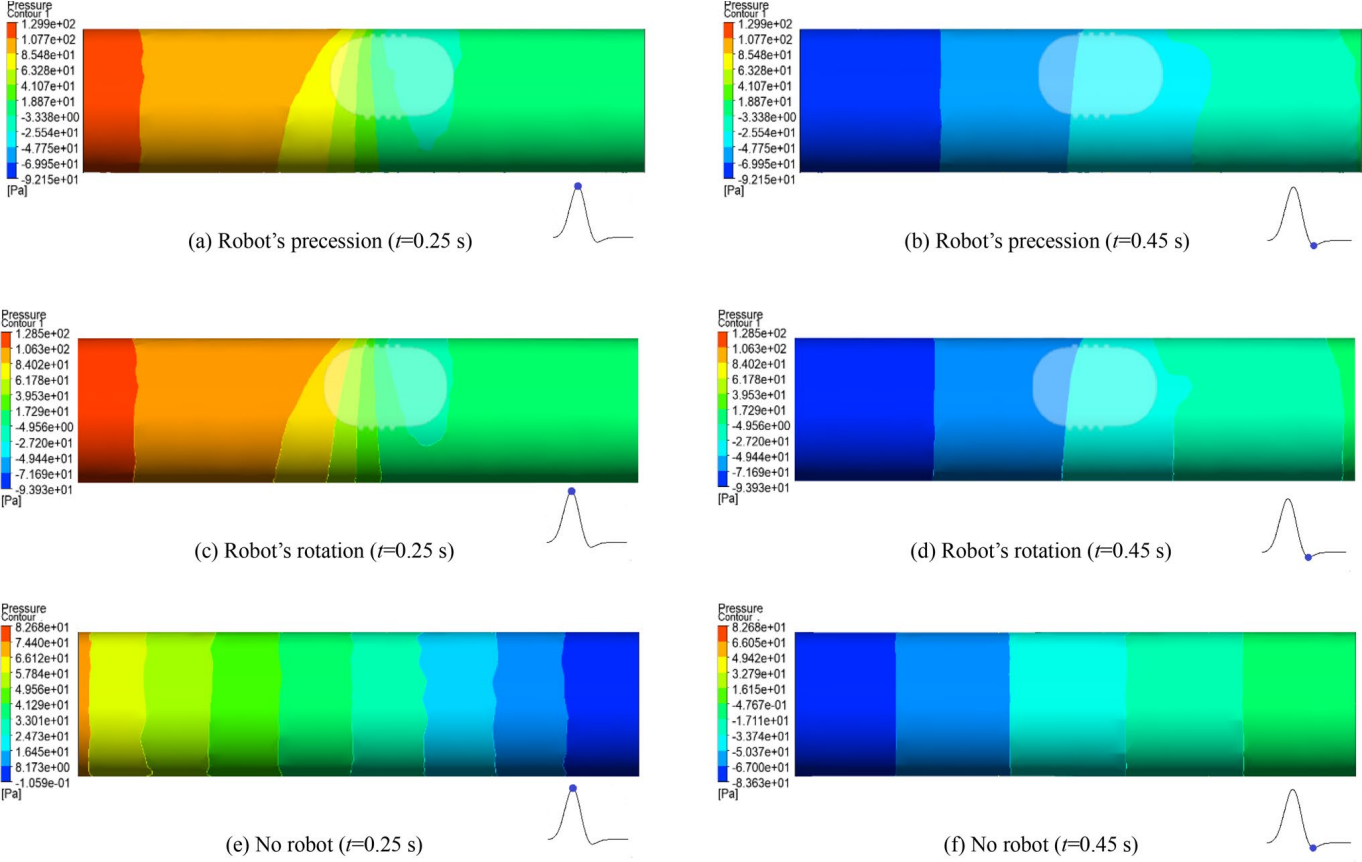
Blood pressure contours under different conditions at 0.25 s and 0.45 s

Figure 8 shows the blood pressure contours at the times of 0.25 s and 0.45 s under the three conditions. At the time of 0.25 s, the changing trends of blood pressure in the three conditions are roughly the same, and the maximum blood pressure at the inlet is continuously decreased along the direction of blood flow. The blood pressure distribution in the blood vessels is relatively disordered during robot’s precession and rotation, especially around the robot where the pressure distribution is more complex. When there is no interventional robot, the blood pressure in the blood vessels is relatively stable. Long-time and complex blood pressure environments can cause excessive proliferation of endothelial cells and thickening of vessel walls. The blood pressure in the blood vessels is roughly the same when the robot is precessing or rotating, with a maximum value of 129.9 Pa, while the maximum blood pressure value is smaller without interventional robots, which is 82.6 Pa. It is indicated that the intervention of the robot has a significant impact on the blood pressure, and the blood pressure is increased by 55.4%, while the impact of the robot operating mode on the blood pressure is minimal when the robot is moving at a low speed of 4 mm/s. During the systolic period, a smaller zone of negative pressure is formed at the tail of the robot in both the robot’s precessing and rotating modes. When there is no interventional robot, the zone of negative pressure of the fluid is located at the end of the blood vessel.

At the time of 0.45 s, the blood pressure distribution under the three conditions is opposite to the trend at the time of 0.25 s, that is, there is a larger zone of negative pressure in the blood vessel, with relatively smaller absolute values. Because the direction of blood flow is changed at the time of 0.45 s, the blood pressure is continuously decreased from the outlet to the inlet, forming a larger zone of negative pressure near the inlet. Long-time negative pressure may cause inflammatory reactions in endothelial cells, and it affects their function and pathological state, even leading to damage and rupture of blood vessels. However, the negative pressure generated during the motion of the interventional robot is within the safe range of the human body, and it will not cause damage to blood vessels.

### von Mises stress of the blood vessel wall

The von Mises stress of the blood vessel wall is influenced by a number of factors. The intervention of a vascular robot alters the internal flow field in the blood vessel, and the von Mises stress of the blood vessel wall is also changed, which may cause damage to the vessel wall or affect the physiological function of the vessel. The higher the von Mises stress of the blood vessel wall is, the greater is the possibility of dissection and rupture of the blood vessel wall at that location. Therefore, the evaluation and monitoring of the von Mises stress of the vessel are of great significance for the prevention and treatment of cardiovascular diseases.

**Fig. 9:**
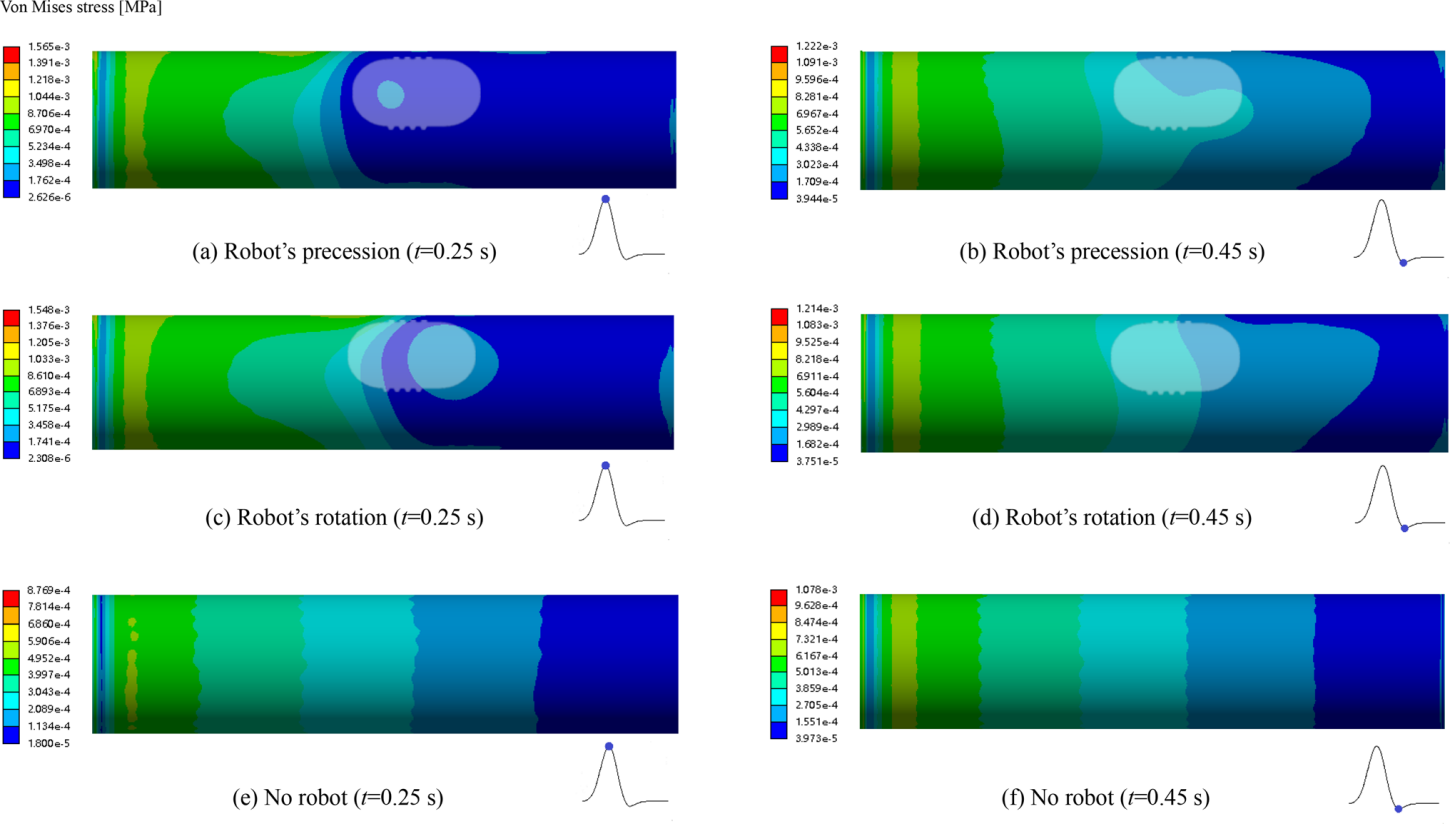
Von Mises stress distribution of the blood vessel wall under different conditions at 0.25 s and 0.45 s

Figure 9 shows the von Mises stress distribution of the blood vessel wall at the times of 0.25 s and 0.45 s under the three conditions. The von Mises stress of the vessel wall is continuously decreased from the inlet to the outlet. When there is an interventional robot, the maximum von Mises stress of the blood vessel wall is 1565 Pa, but for the absence of robots, the maximum von Mises stress is 1078 Pa. The von Mises stress of the vessel wall for an interventional robot is greater than that without interventional robots, the value is increased by 76.2%, and the stress difference of the blood vessels for the robot’s precession and robot’s rotation is relatively small. It is indicated that the use of robots for the interventional therapy in blood vessels will increase the von Mises stress of the blood vessel wall. When the robot moves at a low speed of 4 mm/s, the robot operating mode has little effect on the von Mises stress of the blood vessel wall. For the absence of robots, the von Mises stress of the vessel wall at the time of 0.45 s is greater than that at the time of 0.25 s. In all cases, the von Mises stress of the blood vessel wall is much less than 40 MPa, so the robot intervention is safe and will not cause damage to the blood vessels.

### Deformation amount of the blood vessel wall

**Fig. 10:**
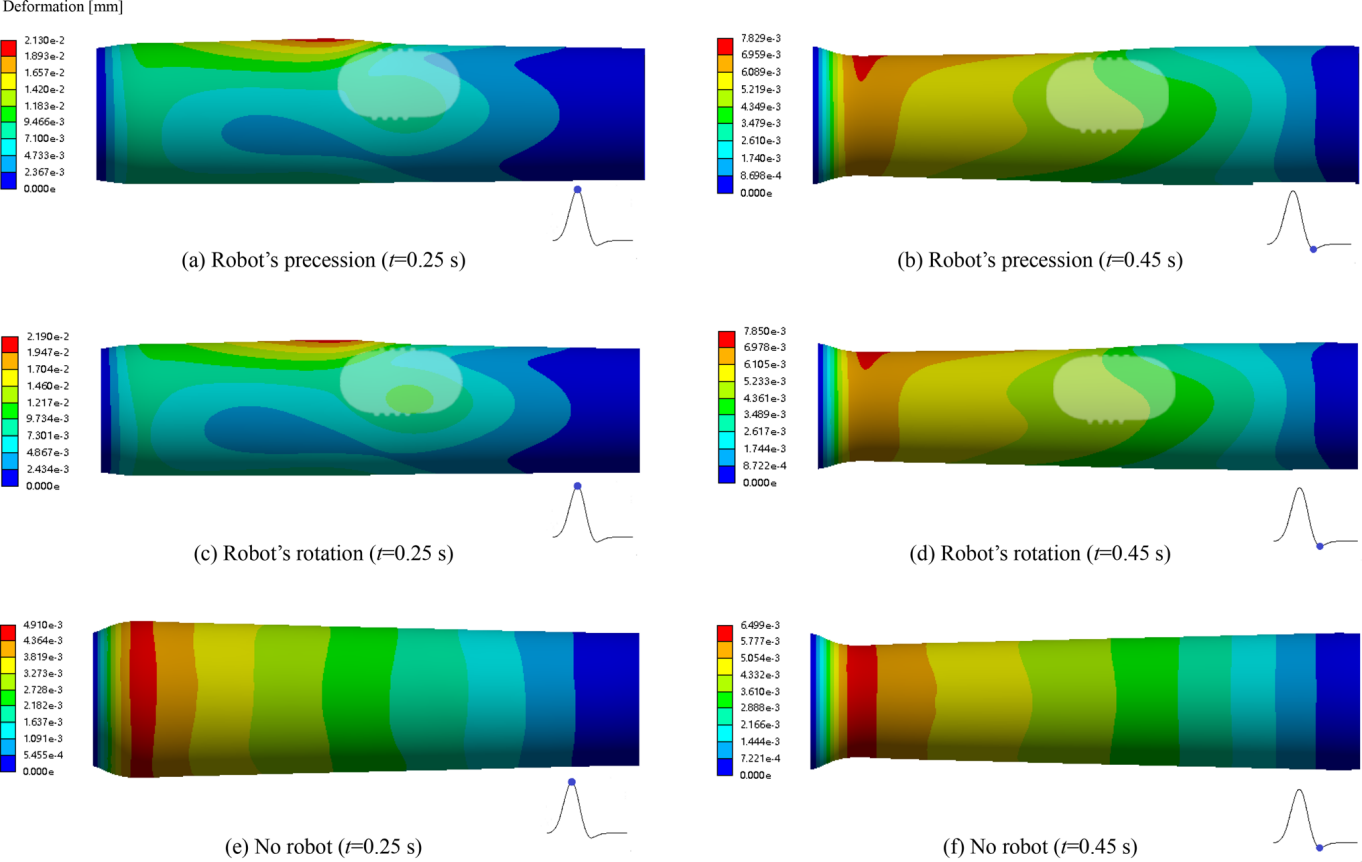
Deformation amount of the blood vessel wall under different conditions at 0.25 s and 0.45 s

Figure 10 shows the deformation amount of the blood vessel wall at the times of 0.25 s and 0.45 s under the three conditions. In order to observe the deformation of various parts of the blood vessel more clearly, the deformation amount of the blood vessel wall was magnified by 190 times to display.

The deformation of blood vessel walls is related to the positive pressure of blood flow on the vessel. The figure shows that when there is an interventional robot, the deformation of the blood vessel wall is greater than that when there is no interventional robot, and the value is almost increased by 346%. Under the low-speed operating conditions of the robot, there is little difference in the deformation amounts of the blood vessel walls between the robot's precession and rotation. When there is an interventional robot, the maximum deformation of the blood vessel wall occurs at the upper front corner of the robot. Because the blood flows to this position and is suddenly blocked, and the blood rapidly flows around both sides of the robot, causing an increase in the wall side pressure and wall deformation at that location. At the same time, the blood flow velocity at the tail of the robot is slow, and the generated side pressure on the wall is relatively small. Due to the boundary conditions set as fixed support boundaries at both ends of the blood vessel, there is no deformation at both ends of the blood vessel. In a cardiac cycle, the maximum deformation of the vessel wall in the three cases is 0.0219 mm, and it is 0.122% of the initial diameter of the vessel. It is indicated that the deformation of the aortic vessel is small, that is, it is difficult to deform.

### Blood flow wall shear stress

The blood flow wall shear stress (WSS) is usually beneficial to the blood vessel wall and can maintain the health and function of vascular endothelial cells. When the WSS value is too high or too small, it can lead to the abnormal function endothelial cells. Therefore, maintaining appropriate WSS is crucial for maintaining the cardiovascular health.

**Fig. 11:**
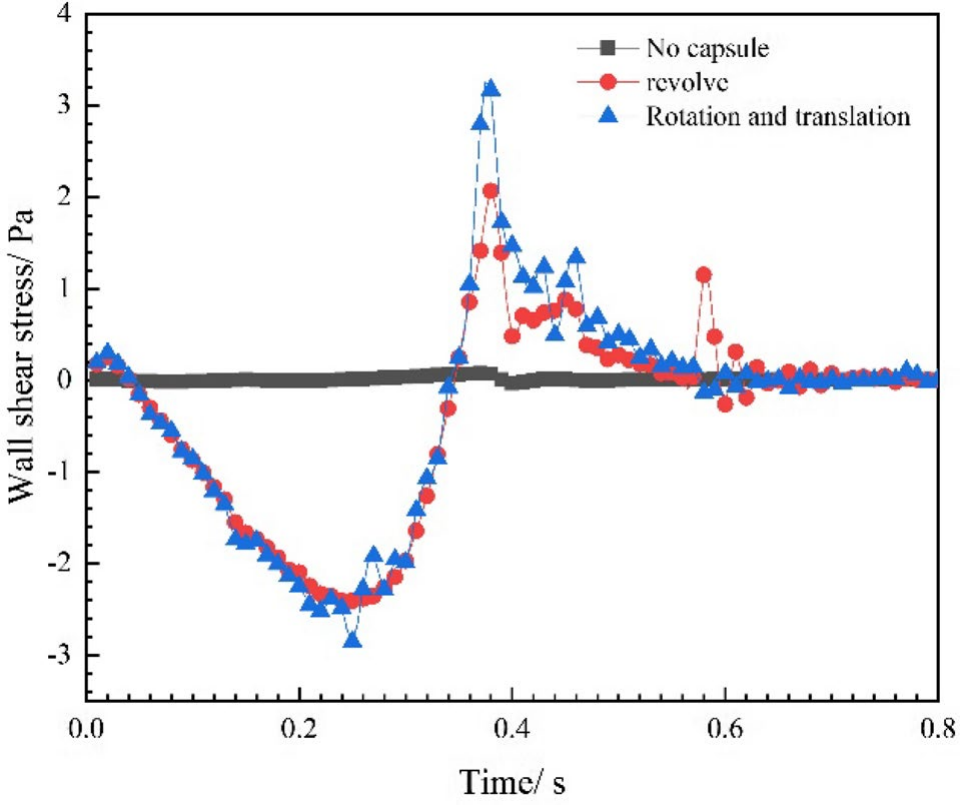
Blood flow wall shear stress under different conditions at different times

The WSS value is related to the flow velocity gradient, which is more difficult to obtain through direct clinical measurements, but it can be solved through the numerical calculation. The WSS values in three different cases were calculated and plotted, as shown in Fig. 11. The positive value of the vertical axis in the figure indicates the direction of WSS to the right, while the negative value of the vertical axis indicates the direction of WSS to the left. Without robot intervention, the maximum WSS is 0.077 Pa. While the WSS value is less than 0.4 Pa for a long time, there will exists insufficient nutrient supply and it is easy to produce atherosclerotic plaques. After the robot is intervened, it makes the WSS values fluctuate within the normal range, which is beneficial to maintain the intravascular health. In the systole, the WSS values approximately equal in the two operating modes after the robot intervention. But in the diastole, the WSS value when the robot is in the precessing mode is slightly larger than that when the robot is in the rotating mode. The variation trend of WSS is basically consistent with the variation law of the blood inlet velocity. During the systole (*t* = 0 ~ 0.25 s), the WSS value is continuously increased and reached its maximum value at the maximum blood flow velocity (*t* = 0.25 s). Within the times of 0.25 to 0.39 s, the WSS value is gradually decreased to 0, and after the direction of WSS is changed, it is continued to increase until it reaches its maximum value (3.17 Pa) at the time of 0.39 s. Within the times of 0.39 to 0.8 s, the WSS value is gradually decreased and fluctuated around 0 after the time of 0.58 s.

In summary, the intervention of a vascular robot results in a significant increase in WSS, while the different operating modes of the robot have little impact on WSS. Therefore, when designing and using vascular robots, it is necessary to consider the force changes on the inner wall of blood vessels to avoid adverse effects on vascular endothelial cells and blood vessel walls.

## Experimental system

Using the PIV technology, the tracer particles are scattered in the fluid flow field, and the transient velocity distribution of the fluid flow field is indirectly measured by measuring the displacement of the tracer particles in a short time interval. For the time interval Δ*t*, a tracer particle in a fluid flow field moves in a two-dimensional plane, and its displacement in the *x* and *y* axial directions is a function of time *t*. The velocity of the fluid at the location of this tracer particle can be expressed as:

6 $$ \left\{ \begin{gathered} v_{x} = \mathop {\lim }\limits_{{t_{2} \to t_{1} }} \frac{{x_{2} - x_{1} }}{{t_{2} - t_{1} }} = \mathop {\lim }\limits_{\Delta t \to 0} \frac{\Delta x}{{\Delta t}} \hfill \\ v_{y} = \mathop {\lim }\limits_{{t_{2} \to t_{1} }} \frac{{y_{2} - y_{1} }}{{t_{2} - t_{1} }} = \mathop {\lim }\limits_{\Delta t \to 0} \frac{\Delta y}{{\Delta t}} \hfill \\ \end{gathered} \right. $$

where *v* is velocity, *t* is time, *x* is the displacement in the *x* axial direction, and *y* is the displacement in the *y* axial direction.

When Δ*t* tends to 0, the velocity obtained from Eq. (6) is the fluid velocity at the point where the tracer particle is located at time *t*_1_. By processing multiple point velocities through a computer, the fluid velocity field in the test plane at time *t*_1_ can be obtained.

**Fig. 12:**
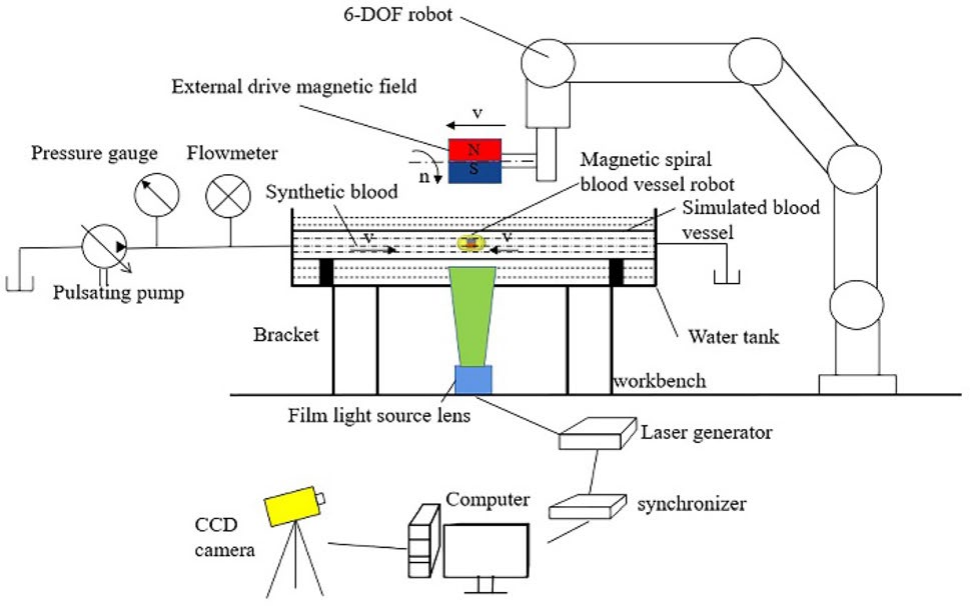
Schematic diagram of the working principle of the experimental system

A PIV measuring system for fluid flow field inside the pipe was designed and constructed when the robot intervened, and the schematic diagram of its working principle is shown in Fig. 12. The high-speed synchronizer controls the laser generator to emit a beam of light (a thickness of 1 mm) at a predetermined time interval, while also controlling the CCD camera to capture images of the zone to be measured. By utilizing the scattering effect of tracer particles on light, the images of the particles during multiple laser exposures are recorded, and the velocity of the tracer particles at each point in the fluid flow field is calculated by the computer, which is the fluid velocity at that point.

Due to the significant difference in densities between the air and the circular elastic pipe, when the sheet-like laser irradiates the surface of the elastic pipe, the laser will refract, and it is difficult to form the same sheet-like irradiation plane inside the pipe, which affects the brightness of the tracer particles and having adverse effects on subsequent image processing, resulting in the deviation from the measurement results. An artificial blood vessel containing an interventional robot is placed in a water tank that is filled with sufficient water to submerge the elastic pipe.

As the sheet-like laser is shined vertically from the bottom of the water tank, there is no refraction. Because of the small difference in densities between water and pipe, the laser hardly refracts when entering the pipe, forming a sheet-like laser surface inside the elastic pipe. The optical axis of the CCD camera is kept perpendicular to the laser plane to reduce the imaging deviation of the tracer particles.

**Fig. 13:**
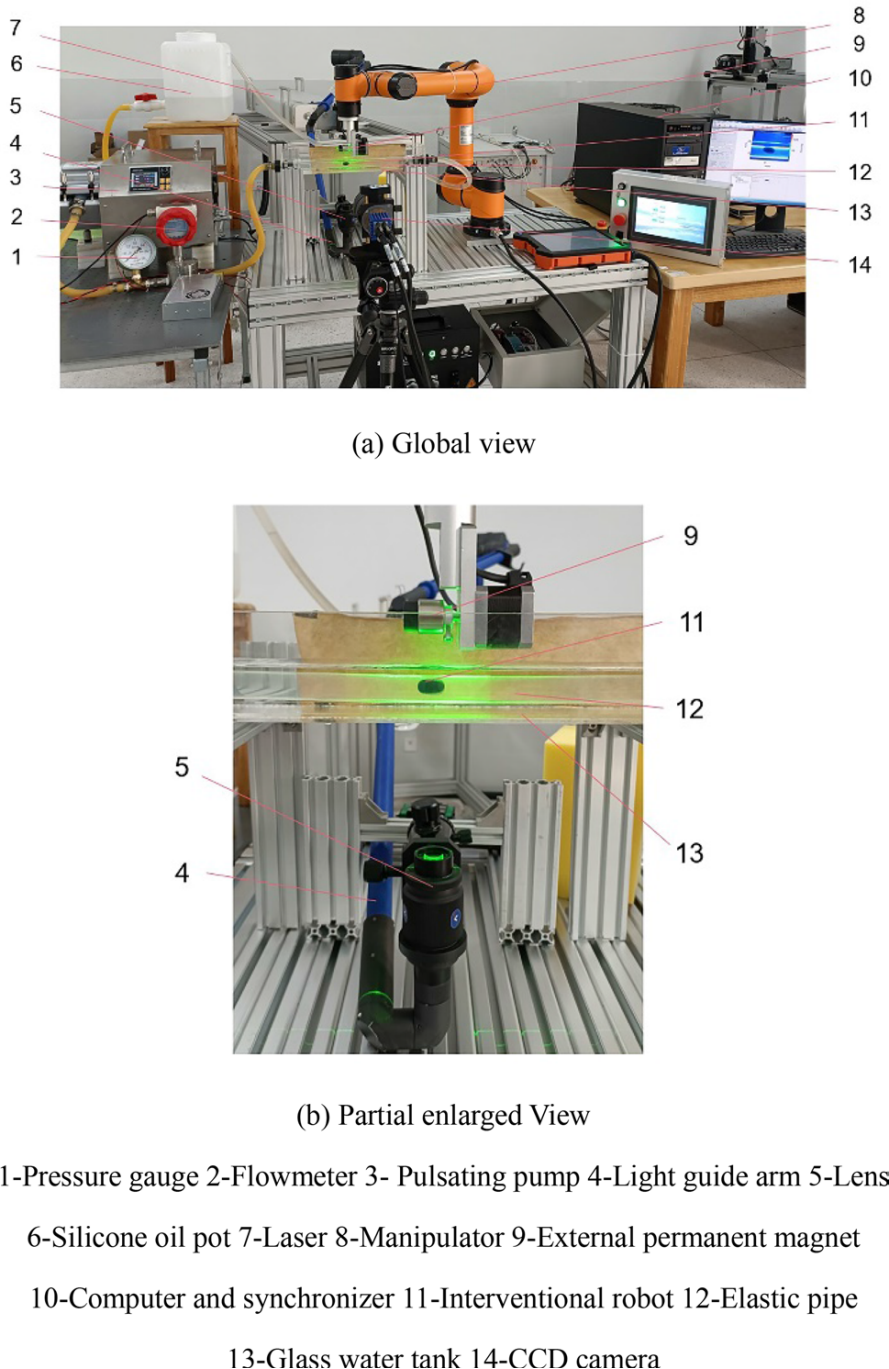
Physical diagram of the experimental system. 1-Pressure gauge 2-Flowmeter 3- Pulsating pump 4-Light guide arm 5-Lens 6-Silicone oil pot 7-Laser 8-Manipulator 9-External permanent magnet 10-Computer and synchronizer 11-Interventional robot 12-Elastic pipe 13-Glass water tank 14-CCD camera

Based on the above working principle, the experimental system consists of a drive module of interventional robots and a PIV measuring module, which is shown in Fig. 13.

The drive module includes a six-degree-of-freedom manipulator, a rotary motor, a worktable, an external permanent magnet, an elastic pipe, and a spiral interventional robot. The pipe has an inner diameter of 18 mm and a length of 80 mm, the material is a flexible plastic, the elastic modulus measured through experiments is about 1 × 10^6^ Pa. The experimental fluid is transparent methyl silicone oil with a viscosity of 5 × 10^–3^ Pa s. The drive module drives the external permanent magnet to translate in the *x*, *y* and *z* axial directions and rotate around its own central axis (*i.e*. *x* axis). In the experiment, the external permanent magnet was set with a translational speed (*v*) of 4 mm/s and a rotational speed (*n*) of 120 r/min, and it drove the interventional robot inside the pipe to perform a precessing motion at the same speed.

The PIV measuring module mainly includes a PIV system, glass water tank, support and computer, etc. The PIV system comes from LaVision Company in Germany, and it consists of an image acquisition and processing system, optical illumination system and tracer particles. The tracer particles are hollow glass beads with a particle size of 8 ~ 12 μm.

The interval time between the two captured images is set to 5 ms. After processing by the DaVis software, the fluid flow field inside the pipe can be displayed. The pulsating flow during the experiment is generated by a peristaltic pump, and in addition, a flowmeter and pressure gauge are connected to the pipe to monitor the fluid data.

**Fig. 14:**
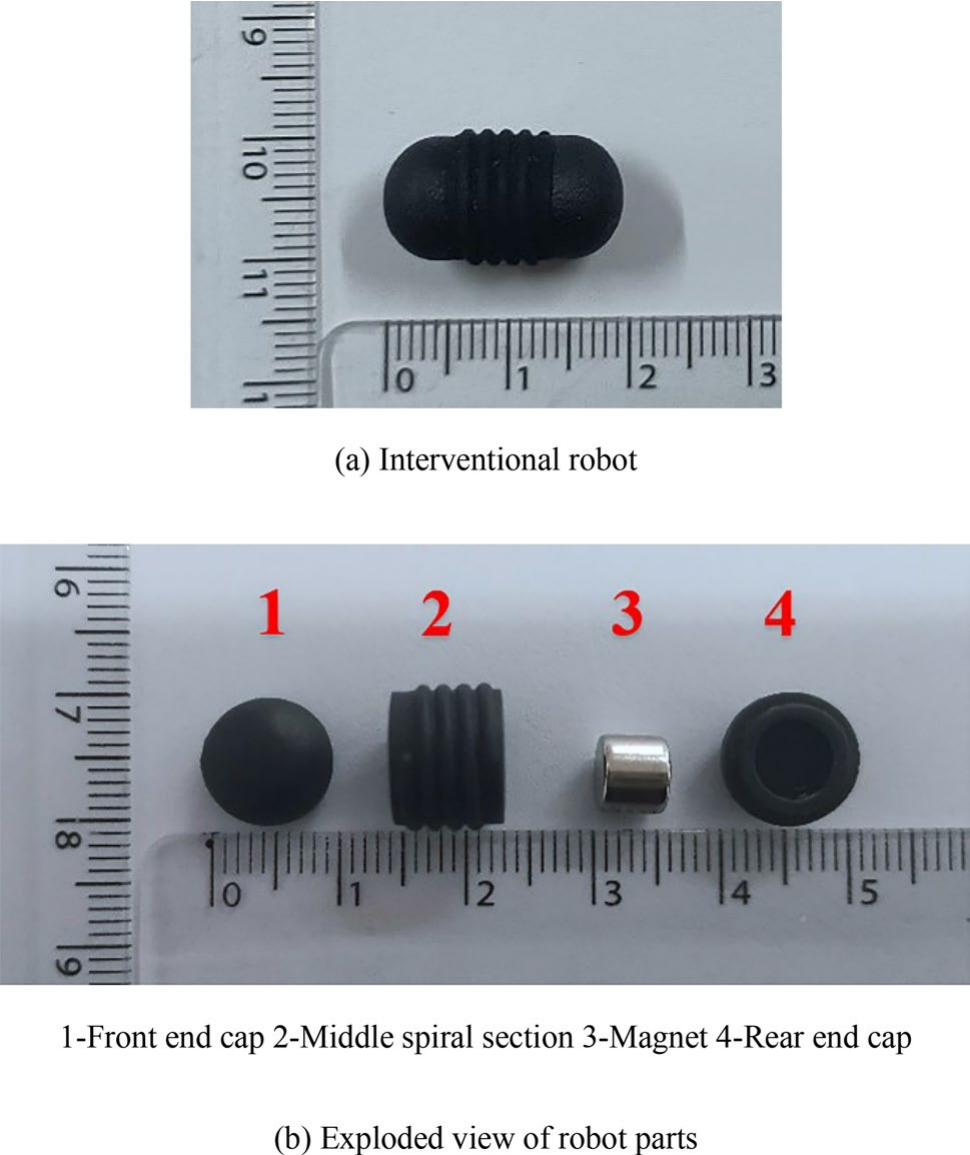
Spiral interventional robot prototype. 1-Front end cap 2-Middle spiral Sect. 3-Magnet 4-Rear end cap

Figure 14 is a prototype of a spiral interventional robot made of bioplastics using 3D printing. The surface is black, with a total length of 18 mm. Both ends are semicircles with a diameter of 10 mm, and the middle section is a cylinder with a length of 8 mm. The surface of the middle section is wrapped with a single head thread, with a spiral shape of a semicircle with a radius of 0.5 mm, a pitch of 1.5 mm, and an effective length of 6 mm. The built-in magnet is a cylinder with a diameter of 6 mm and a length of 5 mm.

**Fig. 15:**
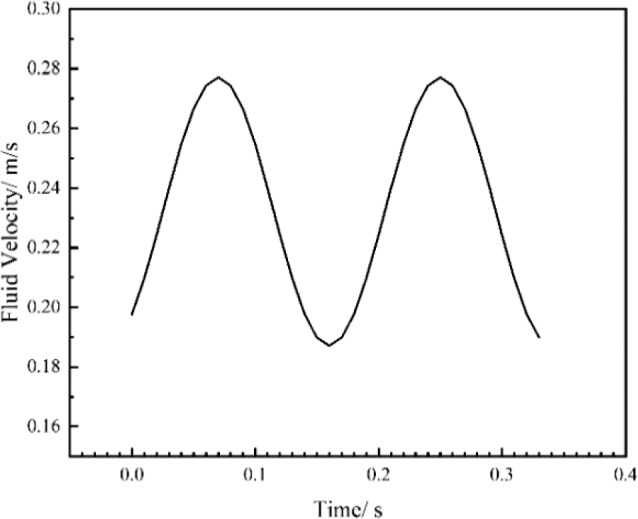
Fitting curve of the output fluid velocity of the peristaltic pump

By combining the flowmeter, the amplitude and frequency of the output fluid velocity of the peristaltic pump are adjusted to make the velocity curve of the output fluid close to the curve near the peak of the blood flow velocity at the aortic inlet in Fig. 6 (marked in red) within a cycle. Figure 15 shows the output fluid velocity curve of the peristaltic pump.

## Experimental results and analysis

Under the condition of pulsating blood flow, the manipulator controls the external permanent magnet to drive the magnetic spiral robot to precess in the pipe at a moving speed of 4 mm/s and a rotational speed of 120 r/min. The relevant parameters for the numerical calculation and the experimental measurement are the same. The pulsating velocity of the fluid generated by the peristaltic pump in the experiment may deviate from Fig. 15, resulting in a deviation from the numerical calculation results. During the precessing motion of the interventional robot, due to the impact of pulsating blood flow, the robot may occasionally produce small shaking, affecting the surrounding blood flow. Though we select the data from the relatively stable operating state of the robot, it will also cause deviations between experimental measurement results and numerical calculation results.

**Fig. 16:**
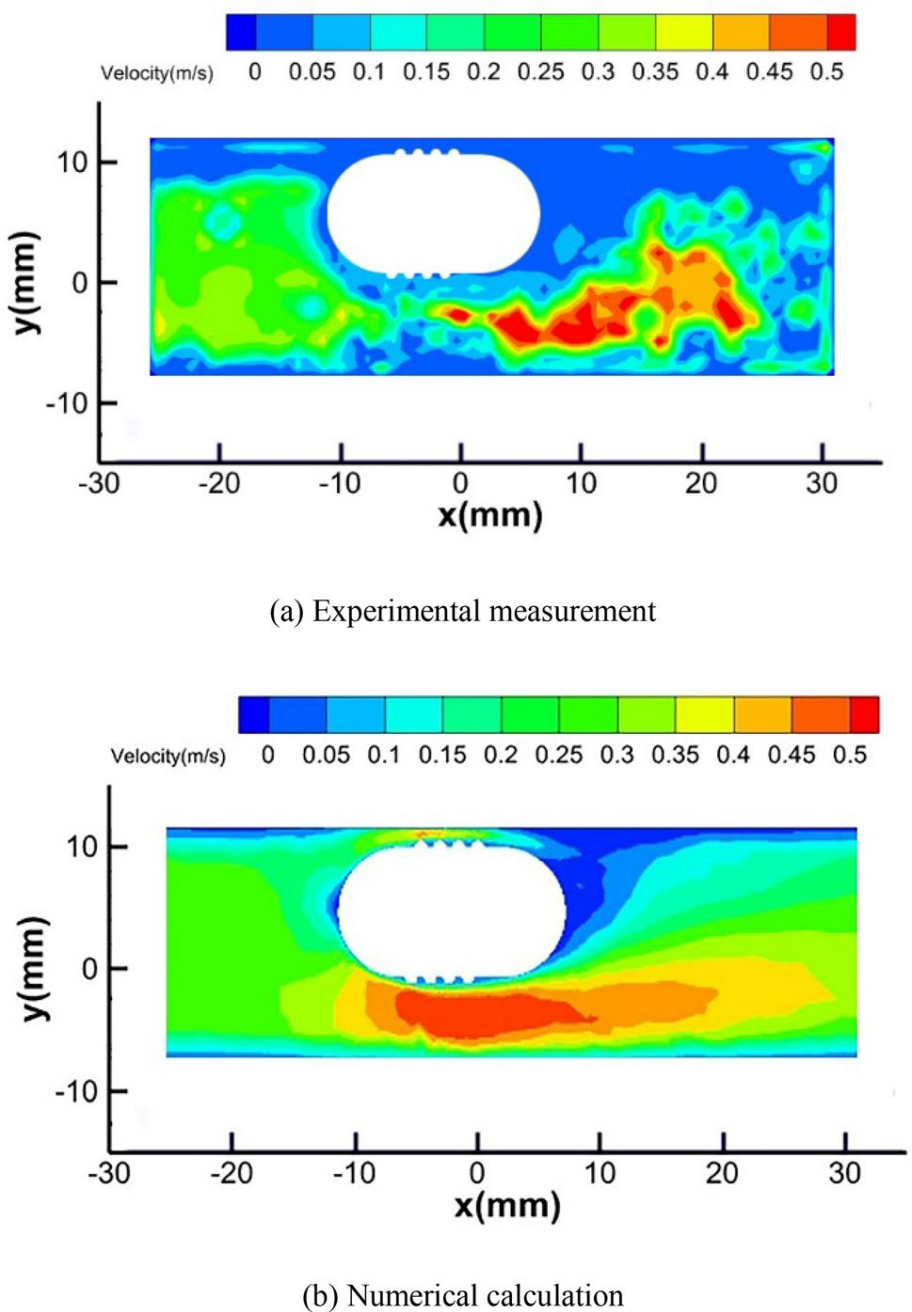
Fluid velocity around the interventional robot (*v* = 4 mm/s, *n* = 120 r/min)

Figure 16 is the comparison between the numerical calculation results and experimental measurement results of the fluid velocity around the interventional robot under the same parameters. The velocity distribution of the fluid around the interventional robot measured by the numerical calculation is basically consistent with that measured by the experiment, which indicates that the numerical calculation is effective. Because of the presence of the robot, the inner diameter of the pipe is reduced, so the velocity of the fluid will be increased when it enters the vicinity of the robot, forming a high-speed zone below it. When the fluid flows through the robot, it will bypass its surroundings and converge at its tail, thus forming two low-speed zones directly in front of and behind the robot.

**Fig. 17:**
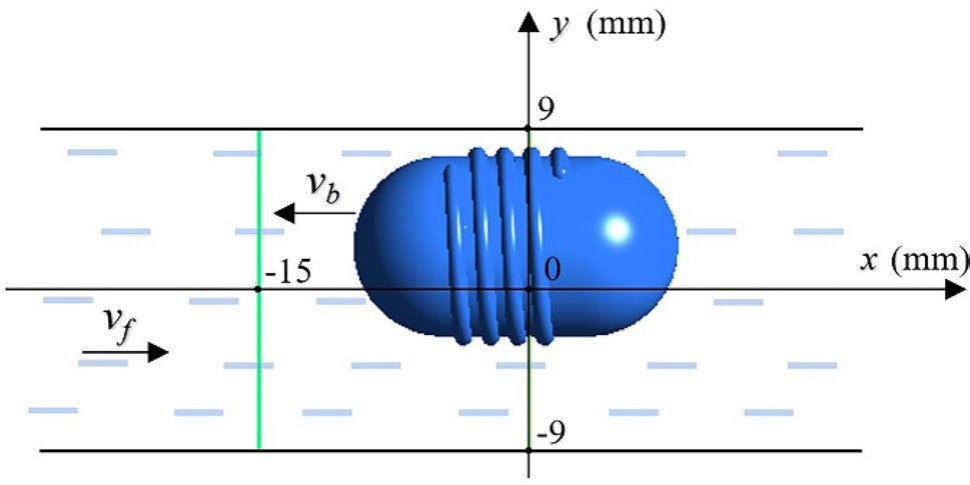
Two positions for comparing calculated and measured results

To further verify the correctness of the numerical calculation, two positions, as shown in Fig. 17, at the center and 15 mm in front of the robot, were selected and the calculation results and measurement results of fluid velocity at these two positions are compared. In the figure, *v*_*f*_ represents the flowing direction of the fluid, and *v*_*r*_ represents the moving direction of the robot.

**Fig. 18:**
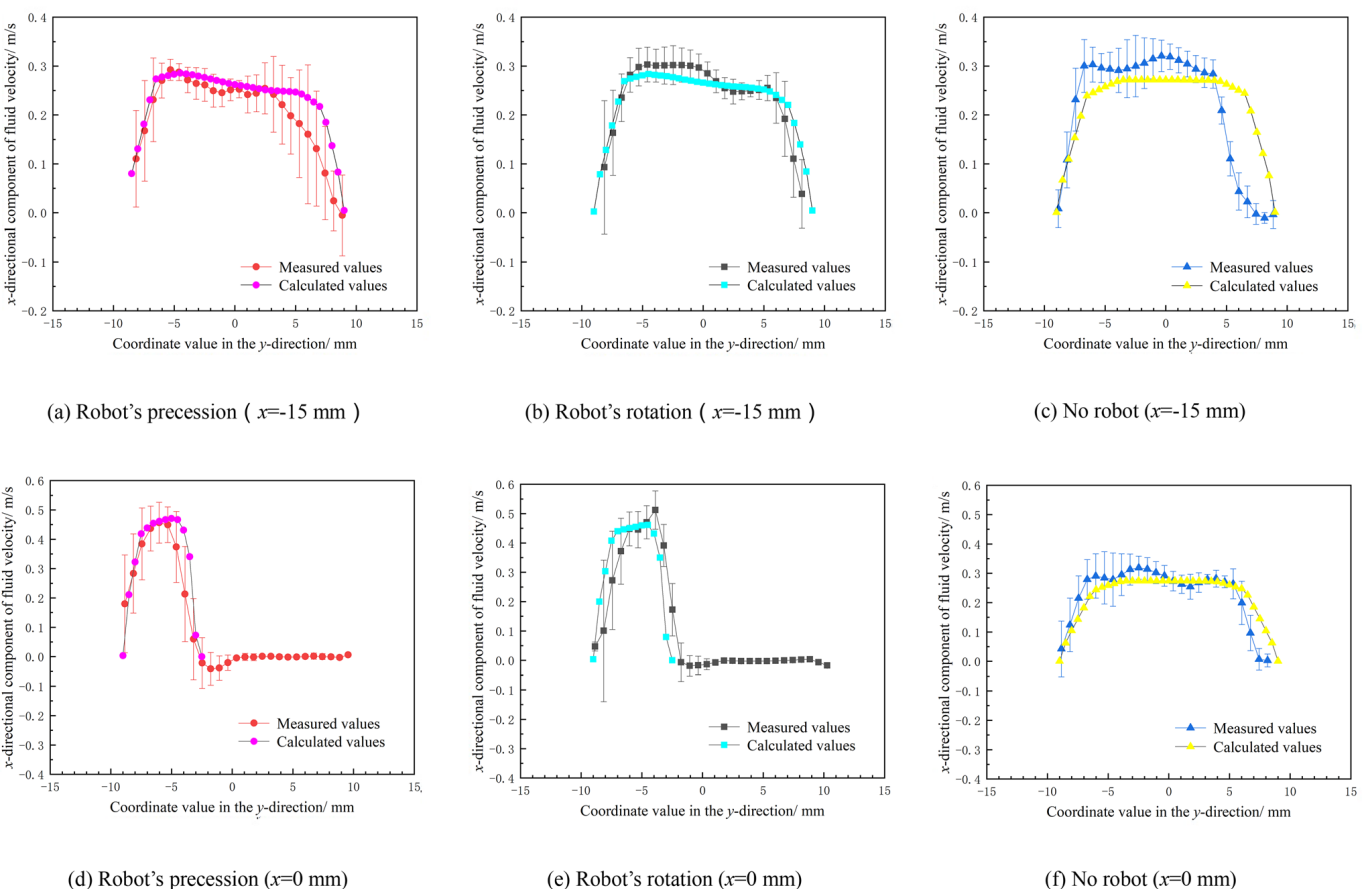
Fluid velocity at different positions around the interventional robot in different states

Figure 18 is the comparison between the numerical calculation results and experimental measurement results of the fluid velocity at the above two positions under three conditions: robot’s precession, robot’s rotation, and no robot. The average values of multiple measurement results are used as the experimental values, and the standard deviations of multiple measurement results are used as the error bars to represent the uncertainty of the measured data.

The figure shows that the changing trends and magnitudes of the fluid velocity obtained from the numerical calculation and experimental measurement in the three cases are basically consistent, and it further proves the correctness of the numerical calculation method in this paper.

## Conclusion

The mutual relationship between blood flow, blood vessel deformation, and interventional robot motion was analyzed based on the bi-directional fluid–structure interaction method. When the robot intervenes into the aorta, the blood flow velocity streamlines when using the CFD method is basically the same as that when using the FSI method, but the values are slightly smaller. We compared and analyzed the differences in changes in blood flow streamline, blood pressure, von Mises stress, and vessel deformation under three cases: robot’s precession, robot’s rotation, and no robot. We also discussed the relationship between blood flow wall shear force and blood flow inlet velocity, and provided a basis for selecting robot operating parameters. It is found that the intervention of the robot has a certain increasing effect on the hemodynamic parameters, and the blood flow rate, blood pressure, equivalent stress and deformation of the vessels are increased by 76.4%, 55.4%, 76.5%, and 346%, respectively, but the values are within the safe range and will not cause the damage to blood vessels. The results show that the intervention of the robot increase the blood flow rate, blood pressure, equivalent stress and deformation of the vessels by 76.4%, 55.4%, 76.5%, and 346%, respectively.

When the robot runs at low speed, its operating mode has little impact on the hemodynamic parameters. A PIV measurement system for the fluid flow field inside the pipe during robot intervention was designed and built. In an elastic pipe, the fluid velocity around the robot was measured under three conditions: robot’s precession, robot’s rotation, and no robot. The results show that the distribution and magnitude of fluid velocity around the robot measured are basically consistent with the computed results, which proves the feasibility and correctness of the numerical calculation method in this paper. In practical applications, with the help of CT and other devices, the approximate location of the diseased blood vessels is obtained. With the assistance of medical imaging equipment, the interventional robot is implanted into the blood vessels remotely from the target location, and is driven by an external magnetic field to move at the target location. During the robot's movement to the target location, the robot's movement will affect the blood flow distribution in the blood vessels, causing changes in hemodynamic parameters. Therefore, it is necessary to study whether the interventional robot will have adverse effects on blood vessels and cause vascular damage while passing through normal blood vessels and working at diseased areas. Our work provides important references for the hemodynamic research and optimization design of the structure and operating mode of intravascular mobile interventional devices during diagnosis and treatment.

## Data Availability

Raw simulation data and materials may be obtained from the corresponding author upon a reasonable request.
